# Tuberculosis vaccine strain *Mycobacterium bovis *BCG Russia is a natural *recA *mutant

**DOI:** 10.1186/1471-2180-8-120

**Published:** 2008-07-17

**Authors:** Peter M Keller, Erik C Böttger, Peter Sander

**Affiliations:** 1 Institut für Medizinische Mikrobiologie, Universität Zürich, Gloriastrasse 30/32, CH-8006 Zürich, Switzerland; 2 Nationales Zentrum für Mykobakterien, Gloriastrasse 30, CH-8006 Zürich, Switzerland

## Abstract

**Background:**

The current tuberculosis vaccine is a live vaccine derived from *Mycobacterium bovis *and attenuated by serial *in vitro *passaging. All vaccine substrains in use stem from one source, strain Bacille Calmette-Guérin. However, they differ in regions of genomic deletions, antigen expression levels, immunogenicity, and protective efficacy.

**Results:**

As a RecA phenotype increases genetic stability and may contribute restricting the ongoing evolution of the various BCG substrains while maintaining their protective efficacy, we aimed to inactivate *recA *by allelic replacement in BCG vaccine strains representing different phylogenetic lineages (Pasteur, Frappier, Denmark, Russia). Homologous gene replacement was achieved successfully in three out of four strains. However, only illegitimate recombination was observed in BCG substrain Russia. Sequence analyses of *recA *revealed that a single nucleotide insertion in the 5' part of *recA *led to a translational frameshift with an early stop codon making BCG Russia a natural *recA *mutant. At the protein level BCG Russia failed to express RecA.

**Conclusion:**

According to phylogenetic analyses BCG Russia is an ancient vaccine strain most closely related to the parental *M. bovis*. We hypothesize that *recA *inactivation in BCG Russia occurred early and is in part responsible for its high degree of genomic stability, resulting in a substrain that has less genetic alterations than other vaccine substrains with respect to *M. bovis *AF2122/97 wild-type.

## Background

Tuberculosis is an infectious disease of enormous global importance. It is estimated that about one third of the human population is latently infected with *Mycobacterium tuberculosis *with 1.6 million people dying annually from the disease [1]. The currently employed tuberculosis vaccine, *Mycobacterium bovis *Bacille Calmette-Guérin (BCG) was originally derived from a virulent strain of *M. bovis *back to 1921, by repeated passages on potato slices soaked in glycerol-ox byle. The primary attenuation is attributed to loss of RD1 locus, which affects a protein secretion pathway [2-5]. Subsequent propagation of BCG in several laboratories around the world resulted in further *in vitro *evolution, which is still ongoing. Genetic alterations are mainly due to deletions and duplications [6], although single nucleotide polymorphisms (e.g. in *mmaA3 *and *sigK*) have also been described [7,8]. The various genetic alterations – some presumably involving RecA-mediated recombination – affect the antigenic, protective, and metabolic properties of BCG. Thus, the term BCG does not refer to an entity but comprises a set of different substrains. Since the early sixties, freeze dried and lyophilized secondary seed lots are used as source of commercially available vaccine strains. WHO vaccine production guidelines [9] call for fresh cultures from secondary seed lots and propose not to exceed twelve passages from the primary seed lot. These recommendations have been modified recently, suggesting not to exceed four passages as phenotypic alterations were observed in vaccine batches undergoing as little as twelve subcultivations [10].

## Results

### Generation of recA knock-out strains

To establish BCG as a vector for stable heterologous antigen expression we intended to stabilize the genome of four BCG substrains representing different phylogenetic lineages by deletion of *recA*. A *recA *phenotype is desirable for live mycobacterial vaccines as homologous recombination is an important contributor of genomic evolution [11,12]. We constructed a replacement vector (Additional file 1) carrying an unmarked, inactive deletion of the *recA *allele. In this vector, a hygromycin resistance cassette and the counterselectable marker *sacB *were cloned downstream of the *recA *allele. Transformation of this kind of vector allows generation of allelic replacement mutants by two subsequent selection steps. In a first step, transformants that have integrated the knock-out plasmid into the bacterial chromosome are selected on hygromycin B containing media. Integration of the vector by homologous recombination, i. e. 3' or 5' of the genomic *recA*, is revealed by Southern blot analyses. In a second step, sucrose-selection is used to select for mutants that have undergone a second intramolecular recombination resulting either in inactivation of the *recA *gene or in reversion to wild-type.

**Figure 1:**
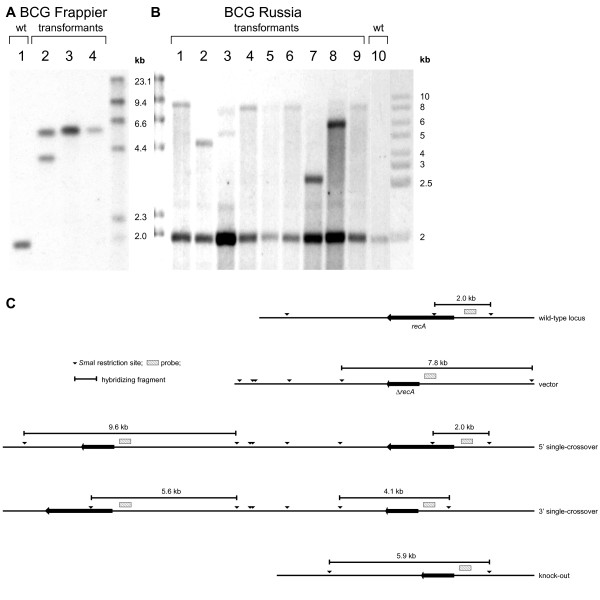
**Southern blot analysis of the *recA *locus**. (A) BCG Frappier. Lane 1, parental *M. bovis *BCG Frappier; lane 2, single-crossover transformant obtained with plasmid pGEM7-*recA*::3xstop-*hsp60*-*sacB*-*hyg*-*aph*; lanes 3-4, *recA *(unmarked) knock-out mutants obtained after counterselection of a single-crossover transformant. (B) BCG Russia. Lanes 1–9 transformants obtained with plasmid pGEM7-*recA*::3xstop-*hsp60*-*sacB*-*hyg*-*aph *resulting from illegitimate recombination; lane 10, parental strain. (C) Schematic drawing of the BCG *recA *locus: the wild-type locus is shown along with the vector used for inactivation, 5' and 3' single-crossover transformant, knock-out mutant.

Most of the transformants obtained in substrains Pasteur, Frappier, and Denmark resulted from homologous integration of the targeting vector (3'or 5') at the *recA *locus (Table 1 and Figure 1A). Subsequent counter-selection of clones arising from homologous recombination events readily resulted in *recA *deletion mutants as demonstrated by Southern blot analyses. In striking contrast, all transformants (16/16 analyzed) of substrain Russia resulted from illegitimate recombination (Table 1 and Figure 1B). Southern blot analysis shows a virtually at random integration of the suicide knock-out plasmid containing the inactivated *recA *in substrain Russia. The presence of a 1.9-kb fragment corresponding to the original *recA *and the presence of a second fragment of varying size indicates non-homologous illegitimate recombination.

**Table 1:** Analysis of recombinants.

BCG substrain	no. of Hyg^R ^colonies	no. of analyzed Hyg^R^	transformants		spontaneously Hyg^R^
				
			homologous recombinants	illegitimate recombinants	
*recA*^+ ^substrains					
Pasteur	54	11	2	3	6
Frappier	90	9	2	1	6
Denmark	377	21	10	2	9
Σ *recA*^+^	521	41	14	6	21
*recA*^- ^substrain					
Russia	72	23	0	16	7

Analysis of recombinants in BCG substrains Pasteur, Frappier, Denmark, and Russia following transformation with *recA *targeting plasmid pGEM7-*recA*::3xstop-*hsp60*-*sacB*-*hyg*-*aph*

### Genomic recA sequence

**Figure 2:**
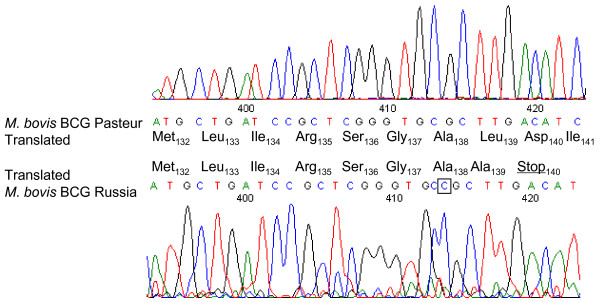
**Comparative alignment**. Comparative alignment of DNA sequencing electropherograms for *M. bovis *BCG Pasteur 1173P2 and BCG Russia displaying the insertional SNP (C_414_) in the *recA *gene of BCG Russia. As a consequence Leu_139 _is changed to Ala, which is immediately followed by a premature stop codon (D140*). Nucleotide and amino acid numbering starts at the *recA *initiation codon.

The absence of marker integration by homologous recombination prompted us to examine the genomic *recA *sequence in BCG Russia in comparison to the annotated sequence of BCG Pasteur 1173P2 [6]. We found a single insertional mutation 413 bp from the *recA *start (Figure 2), which leads to a translational frameshift and a premature stop codon at amino acid position 140 in the major central RecA domain (residues 31–269). The truncated RecA has a length of 139 instead of 790 amino acids and lacks the complete C-terminal part of the protein including loops L1 (residues 156–165) and L2 (195–210) implicated in DNA binding [13]. BCG Frappier, Denmark, and Pasteur *recA *were identical with the annotated reference sequence encoding a functional RecA.

### RecA expression at the protein level

**Figure 3:**
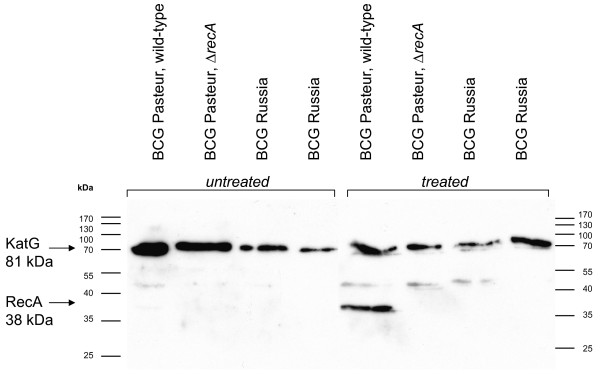
**Western blot analysis**. Western blot analysis of cell-free protein extracts from different BCG preparations (with and without SOS response induction by mitomycin C). Mature RecA protein has an approximate molecular weight of 38 kDa. KatG (81 kDa) served as a loading control. Bands migrating around 50 kDa are unspecific.

Bacteria respond to DNA-damage by coordinated expression of a multitude of genes involved in repair and control of cell division – the SOS response [14]. The SOS response was induced by addition of the DNA-damaging agent mitomycin C (0.2 μg mL^-1^) to a BCG culture, followed by incubation for an additional 24 hours [15]. Western blot analysis using a polyclonal mouse α-RecA antibody demonstrated induction of RecA in strain BCG Pasteur, but absence and failure of induction of RecA in BCG Russia (Figure 3). A genetically engineered BCG Pasteur *recA *mutant (this study) was used as a negative control.

## Discussion

**Figure 4:**
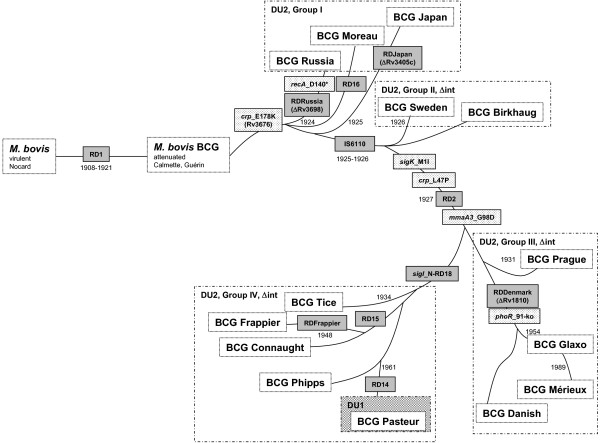
**Genealogy of BCG vaccine substrains**. Genealogy of BCG vaccine substrains, modified from [6] with permission, copyright (2007) National Academy of Sciences, U.S.A., displaying the original virulent ancestor strain *Mycobacterium bovis *(isolated by Nocard in 1908) and the subsequent series of genomic alteration including deletions of regions of difference (RD), single nucleotide polymorphisms, and duplications of genomic regions. The *recA *alteration (*recA*_D140*), the *mmaA3 *point mutation [8], and the 22-bp deletion in *Rv3405c *in BCG Japan [39] have been added to the scheme.

Replication errors, transpositions and recombination events contribute to genetic alterations and drive genome evolution. For BCG various differences in morphology, growth rate, protein expression and genetic make-up have been noted among commercially available substrains [16]. This is presumably a result of the numerous serial passages on natural (potato slices trenched in ox bile) and artificial media, which have led to the acquisition of genomic alterations and further attenuation. Of note, meta-analyses of BCG vaccination trials have indicated protective efficacies ranging from 0–80%. A correlation between the number of serial *in vitro *passages and the decrease in protective efficacy has been observed [17]. Several reasons have been put forward to explain the varying protective efficacies of BCG, among others genetic differences between vaccine substrains as well as within an individual substrain [18]. More recently it has been suggested that the protective efficacy of ancient vaccine strains charcterized by few regions of difference (Figure 4) may be superior to that of the later ones that are more widely used [6]. We have added the newly identified single nucleotide polymorphism to an existent phylogenetic tree. It will be of interest to see whether *recA *is functional in other ancient strains (e.g. BCG Moreau or Japan). Sequencing of *recA *may also help to clarifiy the uncertain origin of the two subcultures of BCG Japan [6].

The RecA-family recombinases have a central role in DNA repair, restoration of stalled replication forks, induction of SOS response, mutagenesis and homologous recombination [19]. In *E. coli*, RecA is involved in several homologous recombination pathways, where it promotes the central steps, i.e. aligning and pairing two DNA molecules, and then promoting a strand switch followed by branch migration [19]. RecA is both ubiquitous and highly conserved among a range of organisms, but variations of the prototypic *E. coli *paradigm exist. *recA *of pathogenic mycobacteria (*M. tuberculosis *complex, *M. leprae*) but not of non-pathogenic mycobacteria (e.g. *M. smegmatis*) is interrupted by an in-frame open reading frame encoding an intein [20]. RecA intein is removed from the precursor RecA by an autocatalytic protein splicing reaction and active RecA, which can complement a *M. smegmatis recA *mutant [21,22] is generated by ligation of amino- and carboxy terminal fragments mediated by intein. *M. tuberculosis *RecA activity differs from *E. coli *RecA activity with respect to single strand DNA-dependent ATP-hydrolysis, co-factor requirement and pH-optimum [23]. Homologous recombination in *M. tuberculosis *complex is sometimes masked by a high degree of illegitimate recombination in certain experiments [24]. The high degree of illegitimate recombination has been attributed to the unusual structure of *recA *[25]. Alternatively, the high degree of illegitimate recombination may be due to the absence of a mismatch repair system [26], which has anti-recombination activity [27]. However, homologous recombination essentially depends on *recA *as demonstrated in a gene conversion assay in a *M. bovis *BCG *recA *mutant [28]. Of note a homologue of *E. coli *RecT, a protein functionally overlapping RecA, is missing in *M. tuberculosis *[29]. Likewise, other recombination genes are also absent from the *M. tuberculosis *genome (e.g. *recE*, *recJ*) [26].

Comparative genomics indicate that recombination events are a major driving force of bacterial evolution [12]. There is extensive evidence for large-scale rearrangements, duplications and deletions resulting from homologous recombination in *M. leprae *[30], *M. tuberculosis *[31] and *M. bovis *BCG [6]. Half of the proteins present in the tubercle bacillus originate from gene duplications [32]. Tandem duplications are generally caused by unequal crossover between homologous sequences or by recombination of short DNA homologies. Homologous recombination between similar sequences may invert or delete genes. Several deletions in the *M. tuberculosis *H37Rv genome resulted from recombination between adjacent repeats of IS6110 elements [31,33]. Sometimes, the molecular mechanisms underlying alterations at particular loci remain obscure and subsequent alterations may mask initial events, e.g. tandem duplication of the DU2 region in *M. bovis *BCG Pasteur arose from duplication of a 100 kb genomic segment that subsequently incurred an internal deletion of 64 kb [18].

## Conclusion

Comparative genome and transcriptome analyses indicate that BCG Russia is an ancient BCG strain most closely related to the original strain attenuated by Calmette and Guérin [6]. However, a key piece of the puzzle was missing. What was the molecular mechanism underlying the high degree of genome conservation in BCG Russia? Here, analysis of the *recA *locus provides a possible clue. RecA, which is the key element of homologous recombination and a driving force in mycobacterial genome evolution but which is not mandatory for conferring protection of BCG in animal models of tuberculosis [11], is disrupted by single nucleotide insertion in BCG Russia. This mutation results in a frameshift and premature translational stop and most probably contributed to the genome stability of this substrain of BCG. Of note genome stability of the obligate endosymbionts of aphids, *Buchnera aphidicola*, is also associated with lack of *recA *[12].

## Methods

### Bacterial strains

*Escherichia coli *DH5α was obtained from Pharmacia (GE Healthcare, Uppsala, Sweden) and was used for plasmid propagation. *M. bovis *BCG Pasteur 1173P2 was obtained from the strain collection of the Institute Pasteur, Paris, France (strain #CIP105050). *M. bovis *BCG Denmark (1331) was obtained in the form of a vaccine production lot through the Statens Serum Institute, Copenhagen, Denmark. *M. bovis *BCG Frappier (Montreal, ATCC #35735) primary lot, dated 1973, lot number 1376141, was obtained from the American Type Culture Collection (ATCC), Rockville, MD, USA. The Frappier strain held by ATCC was initially transferred from the Institut Pasteur to Frappier's Institute in Montreal in 1937, was integrated in the Trudeau Mycobacterial Culture Collection and finally went to the ATCC. A vaccine production lot 547–1104 K. 1491 of M.*bovis *BCG Russia (corresponding to ATCC #35740) was obtained through Medgamal Inc. (TD Allergen, Moscow, Russia). This was the first documented daughter strain distributed by Institut Pasteur in 1924 [34] going directly to Russia. It is referred as an 'early strain' with regards to its genetic characteristics (regions of difference, insertional elements, antigen expression pattern).

The identity of the BCG substrains was confirmed using morphological features (roughling, generation time), PCR amplification and sequencing of described regions of difference as described by [35].

Media, transformation and DNA damage induction. The media for growing *E. coli *DH5α, and substrains of *Mycobacterium bovis *BCG have been described previously [22]. *M. bovis *BCG substrains were revitalised by addition of 1 mL Middlebrook 7H9 medium to a lyophilized batch. For DNA damage induction experiments *M. bovis *BCG was grown in Middlebrook 7H9 medium with addition of 10% (vol/vol) OADC in motionless tissue culture flasks in a 37°C incubator. Published protocols were followed for the preparation of electrocompetent cells of mycobacteria and for electroporation [22]. The *recA *mutants of BCG Pasteur, Denmark and Frappier were generated by allelic replacement using a suicide knock-out vector.

To induce DNA damage, mitomycin C (0.2 μg ml^-1^) was added to 20 mL of growing cultures (at an OD_600 _of 0.6) and incubated for 24 h. Thereafter, bacteria were harvested by centrifugation and prepared for cell lysis.

### Construction of recA knock-out plasmid

Suicide vector pGEM7-*recA*::3xstop-*hyg*-*aph *was generated as follows. A 5.2-kb *Apa*I fragment from plasmid pEJ126 [36] containing the *M. tuberculosis recA *gene was subcloned into the *Pst*I site of plasmid pBluescript-KSII(-) after blunting of both fragments with T4 DNA polymerase, resulting in plasmid pBluescript-*recA*. From this vector a 1.3-kb internal *Pst*I fragment (*recA *bps 504–1758, corresponding to protein residues 169–586) was replaced by a 22-mer triple translation stop insert with an internal *Mun*I restriction site and *Pst*I overhanging ends (Additional file 1). This 22-mer was generated by annealing of dimeric oligonucleotide primers with 5'-phosphorylated ends. Briefly, two complementary 5'-phosphorylated oligonucleotides (Microsynth, Belgach, Switzerland) were mixed in annealing buffer (10 mM Tris-HCl, pH 8.0; 50 mM NaCl; 1 mM EDTA) at equal volumes (resulting in a final concentration of 5 μM per oligonucleotide). The mixture was heated to 96°C for 5 minutes and cooled down to 25°C over a period of 8 hours. The annealing product was diluted 1:10 and used for ligation to result in plasmid pBluescript-*recA*::3xstop.

An *hsp60*-*sacB *construct in form of an *EcoR*V/*BamH*I fragment from plasmid pLO2 [37] was ligated to the *BamH*I/*EcoR*I site of pMV361 [38] to result in pMV361-*hsp60*-*sacB*, wherefrom an *Xba*I/*Xho*I fragment was ligated in the *Xba*I/*Sac*I site of pBluescript-IIKS(-) (Strategene, La Jolla, CA, USA), to result in pBluescript-IIKS-*hsp60*-*sacB*. The later plasmid was digested with *Apa*I and *Nhe*I and a 2514-bp fragment ligated into pGEM7-Zf(+) (Promega, Madison, WI, USA) digested with *Xba*I and *Apa*I, thereby excising 17 bps from the multiple cloning site to result in pGEM7-*hsp60*-*sacB *(5494 bps), expressing *sacB *under the control of the mycobacterial *hsp60 *promoter. A 1.3-kbp *aph *cassette isolated as a *Pst*I fragment from plasmid pUC4K (Pharmacia) was ligated into the *Nsi*I restriction site of pGEM7-*hsp60*-*sacB *to result in pGEM7-*hsp60*-*sacB*-*aph*. Then we introduced a 1.3-kb *hyg *fragment (cut with *Bpu1102*I and *Van91*I, treated with T4 DNA polymerase to create blunt ends) encoding the hygromycin B resistance cassette into pGEM7-*hsp60*-*sacB*-*aph *(cut with SacI and treated with T4 DNA polymerase). The resulting plasmid pGEM7-*hsp60*-*sacB*-aph-*hyg *was cut with *EcoR*V and *Spe*I. We subsequently introduced a 4-kb *recA*::3xstop fragment from pBluescript-*recA*::3xstop (cut with *EcoR*V and *Spe*I), the resulting suicide vector pGEM7-*recA*::3xstop-*hsp60*-*sacB*-*hyg*-*aph *was used for transformation of BCG strains.

### DNA isolation, sequencing and alignment

DNA was extracted from BCG colonies grown on 7H10. The *recA *gene was amplified from genomic DNA (10 ng) by *Pfu *DNA polymerase using primers #1 and #2. The product was cleaned via gel purification and sequenced using primers #1 to #9 (Additional file 2) using an ABI PRISM 310 DNA sequencer (Applied Biosystems, Forster City, CA, USA). The sequences were aligned using Lasergene (DNASTAR, Madison, WI, USA) SeqMan (version 6). The aligned sequence was compared via BLAST software to the *recA *sequence of *M. bovis *BCG substrain Pasteur 1173P2 [6]. Standard techniques were applied for Southern blot analysis. Approximately 500 ng of genomic DNA was digested with *Sma*I and hybridized to a *recA *probe.

### Preparation of cell-free extracts

Untreated and mitomycin-C-treated bacteria were harvested by centrifugation, washed with PBS buffer (pH 7.2) and resuspended in 300 μL PBS containing 0.5% protease inhibitor cocktail (Sigma). Bacteria were lysed in screw-cap tubes in a water immersion (ice-chilled) sonicator (Elma, Singen, Germany), using maximum duty-cycle during 1 h 20 min with a cooling interval every 20 min. The crude lysate was clarified by spinning at 1 000 × g during 10 min.

### Western blotting

Cell-free extracts corresponding to 50 μg total protein (quantification via Bradford assay) were used for Western blot analyses. Following SDS-PAGE in a 13.3% polyacrylamide gel, proteins were blotted to a PVDF membrane using a semi-dry blotter (Biorad, Hercules, CA, USA) at constant current (0.3 A) for 30 min. Equal loading of proteins was confirmed by Coomassie staining, and by blotting with antibodies against the constitutively expressed mycobacterial protein KatG using a polyclonal mouse α-KatG antibody (1:2500). Detection of RecA was carried out using a polyclonal mouse α-RecA antibody (1:1000). Secondary antibody was HRP-conjugated goat α-mouse antibody (1:2500).

### Accession number

DNA sequence of *M. bovis *BCG substrain Russia *recA *has been deposited with GenBank data base under accession EU442641.

## Competing interests

The authors declare that they have no competing interests.

## Authors' contributions

PMK performed the experiments and wrote parts of the paper. PS and ECB designed the study, analysed the results and were involved in writing of the manuscript. All authors read and approved the final manuscript.

## Funding

This work was supported in part by the University of Zurich, the Niedersächsischer Verein zur Bekämpfung der Tuberkulose Lungen- und Bronchialerkrankungen e. V., and Swiss National Science Foundation (Grant 3100A0_120326).

The *M. bovis *BCG Russia was a gift from Medgamal (TD Allergen, Moscow, Russia). The company had no influence in study design; collection, analysis, and interpretation of data; writing of the paper; and decision to submit it for publication.

## Publication notice

A preliminary, non peer-reviewed version appeared online on the Nature Precedings pre-publication server.

## Supplementary Material

Additional file 1 Cloning of *recA *knock-out vector. Additional figure 1 showing the cloning steps of pGEM7-*recA*::3xstop-*hsp60*-*sacB*-*hyg*-*aph *with a 22-bp triple translation stop insert. Click here for file

Additional file 2 *recA *amplification and sequencing primers. Additional table 1 containing the *recA *amplification and sequencing primers. Click here for file
