# Supplementary material for: Tuberculosis vaccine strain Mycobacterium bovis BCG Russia is a natural recA mutant
Source: BMC Microbiol. 2008 Jul 17;8:120. doi: 10.1186/1471-2180-8-120 (PMC2483709; doi:10.1186/1471-2180-8-120)
Supplement: Additional file 1 — Cloning of recA knock-out vector. Additional figure 1 showing the cloning steps of pGEM7-recA::3xstop-hsp60-sacB-hyg-aph with a 22-bp triple translation stop insert. [file 1471-2180-8-120-S1.pdf]

Additional file 1

Cloning of *recA* knock-out vector

Keller et al. Tuberculosis vaccine strain *Mycobacterium bovis* BCG Russia is a natural *recA* mutant.

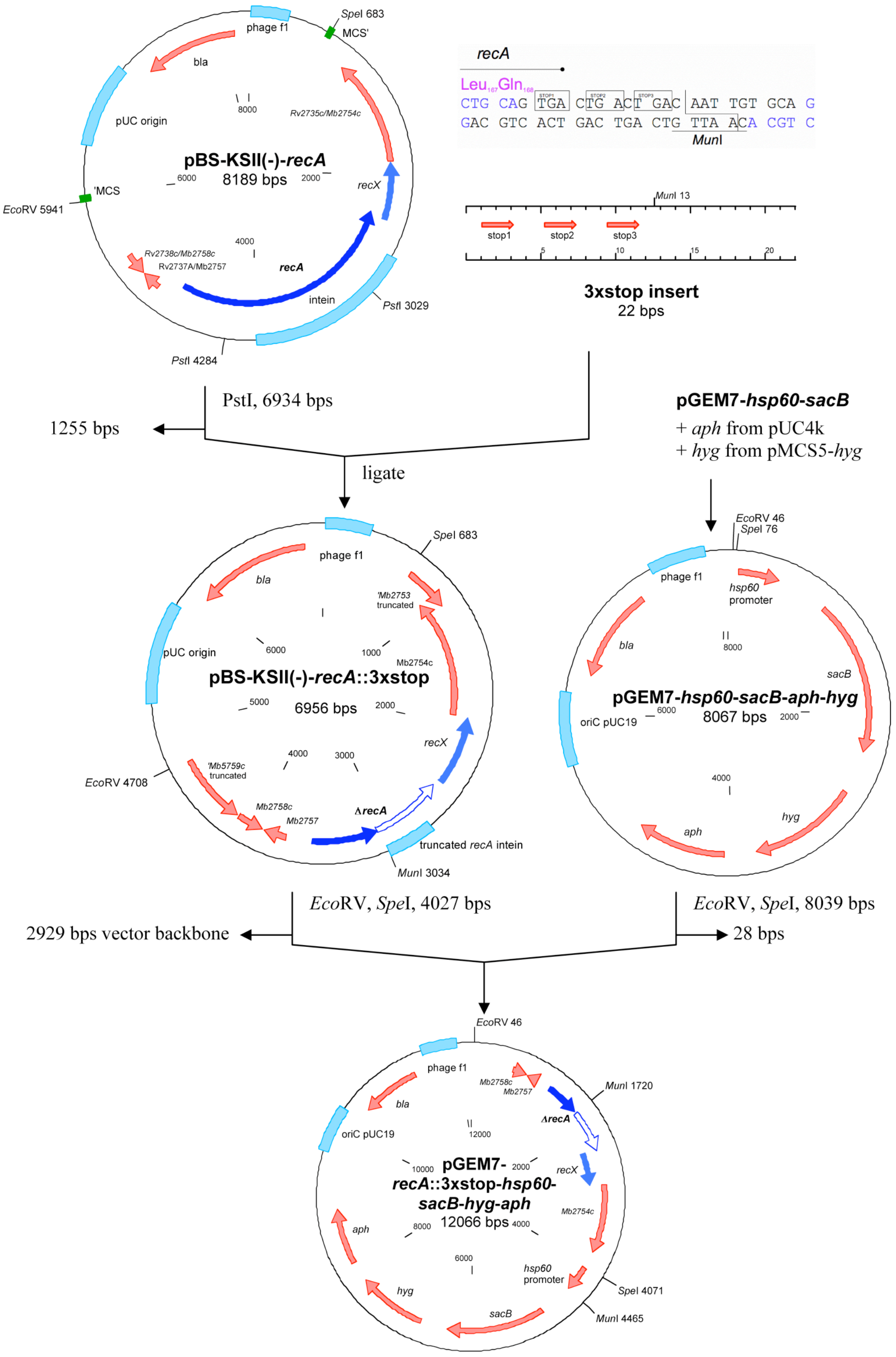

Additional figure 1 - Cloning of pGEM7-*recA*::3xstop-*hsp60-sacB-hyg-aph* with a 22-bp triple translation stop insert.
