# Supplementary material for: Tuberculosis vaccine strain Mycobacterium bovis BCG Russia is a natural recA mutant
Source: BMC Microbiol. 2008 Jul 17;8:120. doi: 10.1186/1471-2180-8-120 (PMC2483709; doi:10.1186/1471-2180-8-120)
Supplement: Additional file 2 — recA amplification and sequencing primers. Additional table 1 containing the recA amplification and sequencing primers. [file 1471-2180-8-120-S2.pdf]

Additional file 2

recA amplification and sequencing primers

Keller et al. Tuberculosis vaccine strain *Mycobacterium bovis* BCG Russia is a natural *recA* mutant.

Additional table 1 - Primer list

Primer #	Descriptor	Sequence 5' to 3'	Usage
1	recA_start	CAT GCA TAT GAC GCA GAC CCC CGA TCG	amplification forward primer, sequencing
2	recA_stop	GCA CTA GTG CCC GCG CCT GCT CTT C	amplification reverse primer, sequencing
3	recA_370_387	GAA CAG GCA CTC GAG ATC	sequencing
4	recA_599_618	TCG GAG TGA TGT TCG GGT CG	sequencing
5	recA_965_983	CCG ATC ACA AGG TGC TGA C	sequencing
6	recA_1307_1326	CCG GTA TCT ACG GCA AGC TC	sequencing
7	recA_1687_1705	GCG CTT ATC CAG GCG ATT C	sequencing
8	recA_1876_1858	CTA AGA CCT GCT TCA TTC C	sequencing
9	recA_1972_1990	TCC GTG ATC CGA GAA GTG C	sequencing
